# Complete Thoracic Ectopia Cordis in Two Lambs

**DOI:** 10.3390/ani14152213

**Published:** 2024-07-30

**Authors:** Liz de Albuquerque Cerqueira, Isabel Luana de Mâcedo, Davi Emanuel Ribeiro de Sousa, Haiane Arruda Luz Amorim, José Renato Junqueira Borges, Fábio Henrique Bezerra Ximenes, Antonio Carlos Lopes Câmara, Márcio Botelho de Castro

**Affiliations:** 1 Veterinary Pathology Laboratory, College of Agronomy and Veterinary Medicine, Universidade de Brasília, Brasília 70910-900, Distrito Federal, Brazil; lizcerqueira@hotmail.com (L.d.A.C.); isabeluanamacedo@gmail.com (I.L.d.M.); daviers@hotmail.com (D.E.R.d.S.); mbcastro@unb.br (M.B.d.C.); 2 Large Animal Veterinary Teaching Hospital, College of Agronomy and Veterinary Medicine, Universidade de Brasília, Brasília 70636-200, Distrito Federal, Brazil; haianearruda14@gmail.com (H.A.L.A.); jrborges@unb.br (J.R.J.B.); fabioximenes@unb.br (F.H.B.X.)

**Keywords:** cardiac anomaly, cleft, congenital defect, malformation, ovine, sheep

## Abstract

**Simple Summary:**

This report characterized complete thoracic ectopia cordis (EC) affecting two newborn lambs from different locations in Brazil, contributing to the knowledge about this rare condition in sheep. Two male newborn lambs presented exteriorized hearts without pericardial coverage, delineated in the thoracic cleft by a fibrous ring of the pericardium and adjacent skin. Clinical findings in the lambs, aside from the EC, were unremarkable. EC is an extremely rare congenital malformation in sheep, with limited cases documented globally. The pathogenesis and etiology remain uncertain, involving potential genetic and environmental factors.

**Abstract:**

Cardiac congenital defects related to inheritance and teratogenesis have been reported in veterinary species and humans worldwide. Among these, ectopia cordis (EC), characterized by an externalized heart through a cleft, is extremely rare in sheep. This report presents the diagnostic features of two cases of complete thoracic EC in newborn lambs. Clinical findings in the lambs, aside from the EC, were unremarkable. Both animals exhibited exteriorized hearts without pericardial coverage, delineated in the thoracic cleft by a fibrous ring of the pericardium and adjacent skin. Histologically, the epicardium was thickened by fibrous tissue in both lambs, with one animal also showing marked edema, hemorrhage, and neutrophilic inflammatory infiltration. The prognosis of EC in the lambs of this study was poor, with fatal outcomes despite attempts at surgical correction.

## 1. Introduction

Malformations in ruminants have been reported worldwide, and expanding the knowledge of congenital defects is required to establish their epidemiology, as well as the most affected breeds and etiology, aiming at prevention. Ectopia cordis (EC) is a congenital abnormal positioning of the heart, which is characterized by cardiac displacement outside the thoracic cavity by a cleft [1]. The condition can be classified according to the position of the ectopic heart into cervical, thoracocervical, thoracic, thoracoabdominal, and abdominal [2], and based on the presence or absence of a pericardial sac covering the heart, as partial or complete, respectively [3].

EC is a rare condition in humans, affecting approximately 0.001% of births [2], and infrequent in cattle [1,4,5,6], dogs [7], rabbits [8], piglets [9], cats [10], and goats [11]. EC is particularly rare in sheep and a poorly documented condition in this species [12,13]. Therefore, this study describes the clinical, diagnostic, and pathological findings in two cases of complete thoracic EC in two lambs.

## 2. Case Series Presentation

Case 1. A 6-day-old crossbred male lamb from a farm in Mossoró County, Rio Grande do Norte State, Northeastern Brazil, was referred to hospital care. The owner reported that the lamb was born unassisted and nursed normally but presented with an “external heart” anomaly (Video S1—Supplementary Material) that had not been observed in other animals on the farm or surrounding flocks. No ultrasonographic evaluation was performed on the dam during pregnancy.

**Figure 1:**
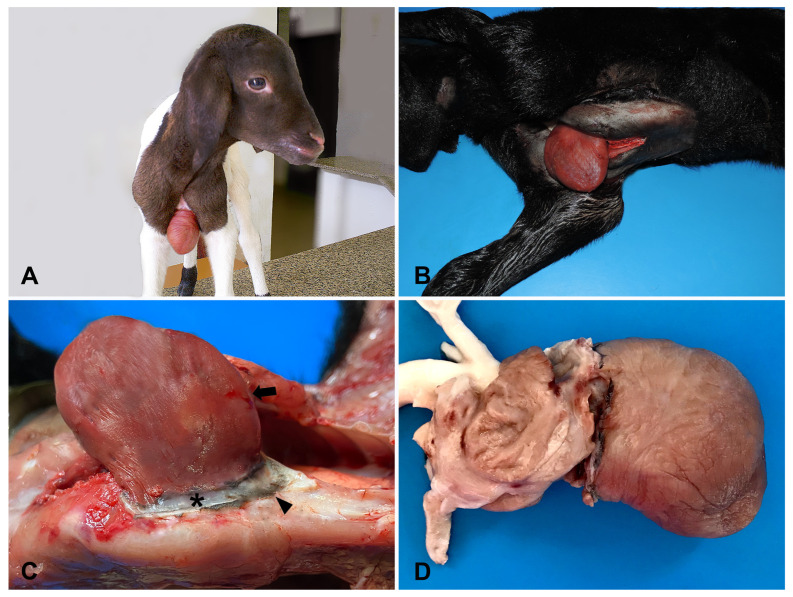
Lambs with ectopia cordis. (**A**). Case 1. A 6-day-old crossbred lamb presenting an externalized heart without pericardial coverage. Case 2. (**B**). Cardiac apex and both ventricles protruding from sternal cleft. (**C**). Epicardium with scattered superficial irregular areas of hemorrhage (arrow) and fibrous pericardium ring (asterisk) surrounded by the remnant skin (arrowhead). (**D**). Mild cardiac constriction with a slightly rounded aspect.

Physical examination revealed the heart protruding from the sternum region without pericardial coverage (Figure 1A), and with clinical parameters (rectal temperature, intestinal motility, heart rate, and respiratory rate) within the reference ranges for the age and species [14]. Hematological (leukogram, erythrogram, and fibrinogen concentration) and biochemical (urea, creatinine, albumin, and globulin levels; aspartate aminotransferase, creatine phosphokinase, and gamma-glutamyl transferase activities) tests yielded unremarkable results (Table S1—Supplementary Material) [15,16]. Cardiac ultrasonography (GE Logiq Pro 100 and a 3.5 MHz convex transducer) revealed no morphological abnormalities in the atria and ventricles. The lamb underwent a surgical procedure to repair the sternal defect, but divulsion of the adhesion between the pericardium band and epicardium caused massive hemorrhage. Hemostasis could not be achieved, resulting in death during surgery.

A midsternal 3.5 × 3.0 cm cleft was identified at the necropsy, from which the majority of the heart was protruding (from the cardiac apex to the coronary sulcus) and adhered to a thick fibrous pericardium band (ranging from 0.3 to 0.7 cm in thickness) transitioning to the surrounding skin. The heart, as well as other organs and tissues, grossly appeared unremarkable.

Samples from organs and tissues were fixed in 10% buffered formalin, routinely processed, embedded in paraffin, and histological sections of 5 µm thickness were stained with hematoxylin and eosin (H&E). A mild fibrous thickening of the epicardium was observed, with no additional microscopic changes noted in the heart or other organs and tissues.

Case 2. A 1-day-old male crossbred lamb, born as part of a twin birth on a farm in Planaltina County, Federal District, Midwestern Brazil, was referred for hospital care. The sheep flock were kept in paddocks with cultivated grass and supplemented with corn silage and commercial feed. The owner reported no similar cases within the flock, and the other twin lamb exhibited no clinical or pathological abnormalities. As in Case 1, no ultrasonographic evaluation was performed on the dam during pregnancy.

**Figure 2:**
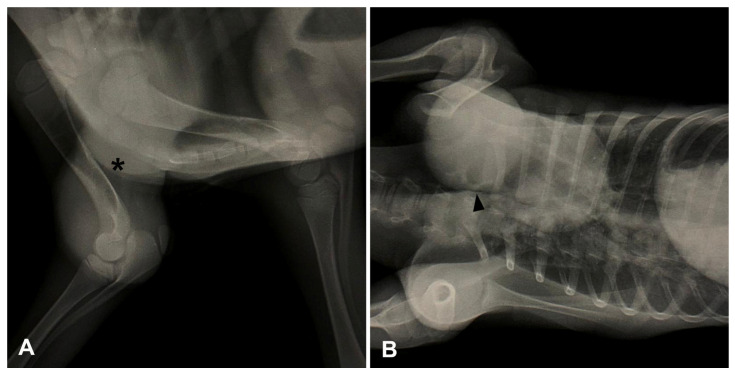
Radiographs of a lamb with ectopia cordis, Case 2. (**A**). Left latero-lateral projection revealing an extrathoracic structure with soft tissue radiopacity (cardiac silhouette) constricted by a fibrous remnant of the pericardium (asterisk), superimposing the thoracic limbs. (**B**). Ventrodorsal projection showing the cardiac silhouette cranial to the 2nd rib on the left side, and the presence of a cleft in the sternum (arrowhead) with poorly defined rib and sternum fusions.

Upon clinical examination, the lamb was alert, active, interacting with its mother and sucking, and the physiological parameters were within the reference values for age and species [14]. The heart was found exteriorized from the body without pericardial coverage (Video S2—Supplementary Material; Figure 1B,C). Radiographic examination revealed an extrathoracic structure with soft tissue radiopacity, compatible with the cardiac silhouette, superimposing the thoracic limbs in the left laterolateral projection (Figure 2A). In the ventrodorsal projection, the cardiac silhouette can be seen cranial to the second rib on the left side, and there is an absence of the first rib on the same side, and the presence of a cleft in the sternum, as well as poorly defined rib and sternum fusions (Figure 2B). An attempt was made to correct the sternal cleft surgically, but the lamb died during the procedure and was subsequently subjected to necropsy.

Grossly, a cleft measuring approximately 3.0 cm × 1.5 cm was observed in the proximal third of the sternal fusion line. Similar to those observed in Case 1, the cardiac apex and both ventricles protruded from the sternal cleft (Figure 1B), with scattered superficial irregular red areas in the epicardium (hemorrhage), transitioning to a fibrous pericardium ring and surrounding skin (Figure 1C). The ectopic heart showed a mild constriction in the area circumscribed by the fibrous ring formed by the remaining pericardial sac (Figure 1D) with a slightly rounded aspect. The right and left atria, aorta, pulmonary trunk, pulmonary veins, and cranial and caudal vena cavae were engorged and filled with blood clots.

**Figure 3:**
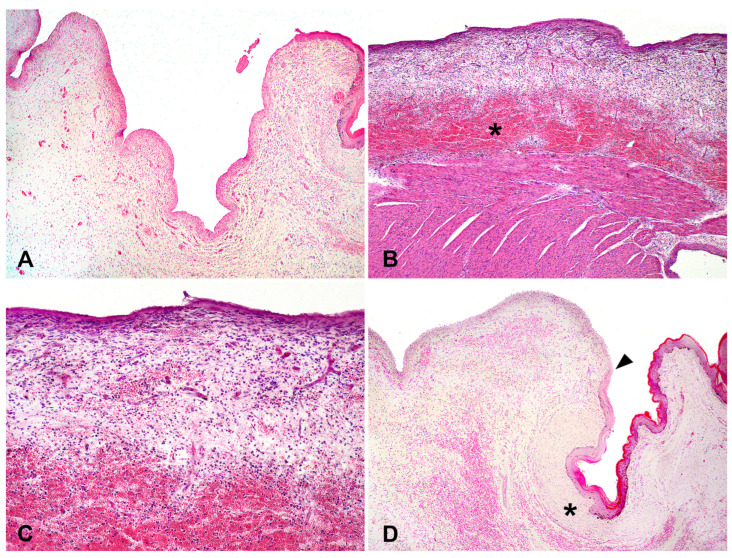
Lamb with ectopia cordis, Case 2. (**A**). Severe thickening of the epicardium (H&E, objective 4×). (**B**). Marked edema and hemorrhage (asterisk) in the epicardium (H&E, objective 10×). (**C**). Inflammatory infiltrate mainly composed of neutrophils (H&E, objective 20×). (**D**). Fibrous pericardial ring (asterisk) and the transition between the pericardium and skin (arrowhead) (H&E, objective 4×).

Histologically, the epicardium exhibits severe thickening (Figure 3A), along with marked edema (Figure 3B), moderate neovascularization and hemorrhage, and infiltration of neutrophilic inflammatory cells (Figure 3C). Additionally, a continuity was observed between the epicardium, fibrous pericardial ring, and skin (Figure 3D). Microscopic findings in other organs and tissues were unremarkable. Both cases of ectopic hearts in lambs herein were classified as complete thoracic EC.

## 3. Discussion

Cardiac malformations have been widely documented in both domestic animals and humans. Among these, EC stands out as an uncommon congenital abnormal position of the heart that is extremely rare in sheep [12,13]. In a study involving 4417 lambs, the incidence of congenital cardiac defects was estimated at 0.1%, with ventricular septal defect being the most frequently observed malformation [17]. Besides cases of ventricular septal defect and EC, multiple cardiac anomalies have also been described in a 12-h-old lamb [18] and associated with diprosopia [19]. In ruminants, the most common cardiovascular congenital anomalies are valvular hamartomas, patent foramen ovale, ventricular septal defect, and transposition of the aorta and pulmonary artery [20].

In both lambs with EC in this study, no specific clinical signs other than cardiac exteriorization were detected, similar to those reported in a lamb that survived six months after birth with the same malformation in India [13]. In contrast, a 5-day-old lamb with EC in Brazil exhibited mild tachycardia and tachypnea, dehydration, apathy, pale mucous membranes, a filiform pulse, hypothermia, and subsequently died [12]. Non-specific clinical signs such as tachycardia and tachypnea were also observed in cattle and goats with EC, with death occurring between ten minutes and two days after birth [5,11]. In cattle with cervical EC, the time of death ranged from three minutes to over ten months after birth [4]. As observed in the two lambs from this study, EC has a guarded prognosis and clinical signs may be absent or variable and are not always associated with cardiovascular impairment.

The cardiac anomalies identified in both lambs were classified as complete thoracic EC, similar to the other two cases in sheep [12,13]. In cattle, cervical EC is the more frequent presentation [5,6,21], contrasting with the predominance of thoracoabdominal EC in cats [10], piglets [9,22], and dogs [7,23]. Thoracic and thoracoabdominal EC are the most common forms in humans and may be associated with the Pentalogy of Cantrell [24,25], which encompasses multiple congenital anomalies alongside ectopic heart positioning [26].

The first lamb of this study showed no gross cardiac changes or other cavitary organ alterations, as previously reported in a lamb with EC from India [13]. In the second lamb, the slightly rounded exposed heart and multifocal random hemorrhagic areas on the epicardium were also described in a case of EC in a Brazilian lamb [12]. In addition, abnormal formation of arteries at the cardiac base [1] and double cardiac apices [1,4,5] were observed in cattle with EC. Other cardiac anomalies, such as ventricular and atrial septal defects, Tetralogy of Fallot [27], and Pentalogy of Cantrell [26], have also been reported in humans with EC.

Histopathological findings such as thickening of the epicardium (Cases 1 and 2), edema, neovascularization, hemorrhage, and neutrophilic inflammatory infiltration (Case 2) have been previously reported in a goat with complete thoracic EC [11]. Generally, reports of EC in cattle are associated with the absence or presence of pericardium coverage and lack of other histological changes [5], such as those found in humans [24,25] and dogs [23]. Histopathological evaluations are infrequent in cases of EC in animal species, as most reports are based on gross evaluations alone [6,12,13,22]. The microscopic findings herein are likely related to an acute inflammatory response due to the exposure of the heart to environmental contaminants without pericardial coverage.

The pathogenesis and etiology of EC remain uncertain and may involve a complex interaction of genetic and environmental factors, although the exact cause is still unknown in many cases. Congenital anomalies of cardiac development during embryogenesis, such as the failure of maturation of mesodermal components in the midline of the thorax and abdomen, may be related to the development of EC [3]. Additionally, mutations in bone morphogenetic protein (BMP) genes, specifically BMP2 and BMP4, which are known to influence the formation and differentiation of cardiac cells during embryonic development, can apparently disrupt the normal development of cardiac anatomy, resulting in defects such as EC [28,29]. Another hypothesis suggests that EC may be a consequence of amniotic band syndrome, typically due to early rupture of the yolk sac, leading to the formation of fibrous bands that interfere with the fusion of the midline thoracic wall [29]. Furthermore, EC may also develop as a result of chromosomal defects such as 16 and 18 trisomy [3,5,30,31].

Experimental studies in rat models have suggested that exposure to teratogenic compounds during embryological development plays a crucial role in the development of EC [8,32]. In Brazil, teratogenic defects have been associated with the consumption of toxic plants, such as *Mimosa tenuiflora*, *Cenostigma pyramidale*, *Prosopis juliflora*, *Conium maculatum*, and species of the genus *Nicotiana* spp., which contain quinolizidine and piperidine alkaloids that reduce fetal movements, thereby inducing congenital deformities in the fetus [33,34]. These toxic plants are associated with craniofacial bone anomalies, ocular malformations, arthrogryposis, and abortions in ruminants, but not with EC [35,36]. As none of these plants were detected in the paddocks at both farms, teratogenic plants were ruled out as the cause of EC in the two lambs from this study.

Teratogenesis associated with viral infections such as Cache Valley, Schmallenberg, Akabane, and Bluetongue Virus can lead to central nervous system and musculoskeletal malformations in ruminants worldwide [37,38,39,40]. These infectious agents have not been observed affecting sheep in Brazil and are considered uncommon, and they have not been associated with cardiac or thoracic cavity malformations [41]. Both lambs with EC in this study were isolated cases, with no other animals detected with malformations in the flocks.

Surgical correction of EC is currently considered the treatment of choice [42,43,44]. The surgical approach is staged, and the primary goals are: (1) returning the heart into the thoracic cavity and (2) reconstruction of the associated sternal defect [42]. Both stages are challenging, and surgical complications include infection, cardiorespiratory embarrassment from direct cardiac compression, and cardiac failure, particularly when associated with intracardiac anomalies [43,44]. Therefore, the success of surgical treatment is multifactorial, with the type of ectopia and other concomitant malformations potentially influencing the prognosis [22,24,42,43,44]. Even in humans, the thoracic form of EC is considered uniformly fatal, with a review of 91 cases having only two children surviving beyond the first year of life [42]. Despite that, complete thoracic EC with a complete sternal cleft was successfully corrected using a novel application of a mandibular distraction device in a 10-week-old child [43]. In livestock, surgical correction of EC is rarely a viable option due to economic and practical constraints, leading to a generally poor prognosis and a high mortality rate [6,12]. In the lambs herein, the vigorous adhesion of the epicardium to the pericardial band caused fatal hemorrhage during divulsion. Perhaps, in future cases of complete thoracic EC without other congenital anomalies, the most appropriate approach would be a skin flap closure over the exposed heart to reduce the risk of infection, since there is a report of long-term survival of a cow with cervical ectopia cordis [21]. Nevertheless, as in humans, complete thoracic EC has a poor prognosis [42,43], and perhaps the best approach to livestock with this congenital defect would be euthanasia to avoid suffering.

## 4. Conclusions

EC is an extremely rare congenital malformation in sheep, with limited cases documented globally. The pathogenesis and etiology remain uncertain, involving potential genetic and environmental factors. In Brazil, no specific viral infections or toxic plants have been definitively linked to EC in sheep. Further research is essential to understand the underlying causes and to prevent the increase in the prevalence of this rare malformation in flocks.

## Data Availability

Data are contained within the article or Supplementary Materials.

## Supplementary Materials

The following supporting information can be downloaded at https://www.mdpi.com/article/10.3390/ani14152213/s1: Supplementary Material includes two video files, and one table file. Table S1: Hematological and biochemical findings in a 6-day-old crossbred male lamb (Case 1) presenting complete thoracic ectopia cordis; Video S1: 6-day-old lamb (Case 1).; Video S2: 1-day-old-lamb (Case 2).
