# Supplementary material for: Complete Thoracic Ectopia Cordis in Two Lambs
Source: Animals (Basel). 2024 Jul 30;14(15):2213. doi: 10.3390/ani14152213 (PMC11310945; doi:10.3390/ani14152213)
Supplement: Supplementary file 1 [file animals-14-02213-s001.zip › animals-3122725-supplementary.pdf]

Table S1. Hematological and biochemical findings in a 6-day-old crossbred male lamb (Case 1) presenting complete thoracic ectopia cordis.

Parameter	Case 1	Reference values*
Hematocrit (%)	35	24-50
Red blood cells ($10^6/\mu L$)	8.5	8-16
Hemoglobin (g/dL)	10.2	8-16
Total plasma protein (g/dL)	6.4	6-7.9
Albumin (g/dL)	2.6	2.4-3
Globulin (g/dL)	3.8	3.5-5.7
Fibrinogen (mg/dL)	200	100-500
Total leukocytes(/µL)	5.5	4,000-12,000
AST (U/L)	194	60-280
CK (U/L)	156	73-236
GGT (U/L)	43	20-52
Creatinine (mg/dL)	1.2	1.2-1.9
Urea (mg/dL)	20	17.1-42.8

AST: aspartate aminotransferase; CK: creatine phosphokinase; GGT: gamma-glutamyl transferase. * Meyer & Harvey [15], Kaneko et al. [16].

References

15. Meyer, D.J.; Harvey, J.W. *Veterinary Laboratory Medicine: Interpretation and Diagnosis*, 2nd ed.; Saunders: Philadelphia, PA, USA, 2004; 351p.

16. Kaneko, J.J.; Harvey, J.; Bruss, M. (Eds.) *Clinical Biochemistry of Domestic Animals*, 6th ed.; Academic Press: San Diego, CA, USA, 2008; 928p
